# Author Correction: Stable isotope evidence for pre-colonial maize agriculture and animal management in the Bolivian Amazon

**DOI:** 10.1038/s41562-025-02101-z

**Published:** 2025-01-08

**Authors:** Tiago Hermenegildo, Heiko Prümers, Carla Jaimes Betancourt, Patrick Roberts, Tamsin C. O’Connell

**Affiliations:** 1 isoTROPIC Research Group, Max Planck Institute of Geoanthropology, Jena, Germany; 2 Laboratory of Tropical Archaeology, Museum of Archaeology and Ethnology, University of São Paulo, São Paulo, Brazil; 3 German Archaeological Institute, Commission for Archaeology of Non-European Cultures, Bonn, Germany; 4 Department for the Anthropology of the Americas, University of Bonn, Bonn, Germany; 5 Department of Archaeology, University of Cambridge, Cambridge, UK

**Keywords:** Archaeology, Stable isotope analysis, Agriculture, Archaeology

Correction to: *Nature Human Behaviour* 10.1038/s41562-024-02070-9, published online 23 December 2024.

In the version of this article initially published, the surname of Tiago Hermenegildo was misspelled (Hermengildo) and has now been amended in the HTML and PDF versions of the article.

