# Supplementary material for: Stable isotope evidence for pre-colonial maize agriculture and animal management in the Bolivian Amazon
Source: Nat Hum Behav. 2024 Dec 23;9(3):464–71. doi: 10.1038/s41562-024-02070-9 (PMC11936833; doi:10.1038/s41562-024-02070-9)
Supplement: Supplementary file 1 — Supplementary Figs. 1–4, Tables 1–7, and description of sites, samples and chronologies. [file 41562_2024_2070_MOESM1_ESM.pdf]

nature human behaviour

Supplementary information https://doi.org/10.1038/s41562-024-02070-9

Stable isotope evidence for pre-colonial maize agriculture and animal management in the Bolivian Amazon

In the format provided by the authors and unedited

Table of contents

Site context, chronologies and funerary practices **2**

Mendoza site **2**

Salvatierra site **3**

Fauna **5**

References **7**

Figure S1 Radiocarbon Dates from Mendoza site arranged by phase. **9**

Figure S2 Radiocarbon Dates from Salvatierra site arranged by phase. **10**

Figure S3 Boxplots presenting the human $\delta^{13}C$ and $\delta^{15}N$ values from sites in the Amazon, Llanos de mojos and Mesoamerica **11-12**

Figure S4 Boxplots presenting the $\delta^{13}C$ and $\delta^{15}N$ values of the muscovy duck population from Salvatierra and Panama sites **13**

Figure S5 Aerial view from the village of Casarabe, Bolivia **14**

Figure S6 The Mendoza site plan **15**

Figure S7 The Salvatiera site plan. **16**

Table S1 Results of pairwise comparisons (p values) of the $\delta^{13}C$ and $\delta^{15}N$ fauna values. **17**

Table S2 Results of the Tuckey HSD test (p values) of the human $\delta^{13}C$ values according to phase **17**

Table S3 Description of the $\delta^{13}C$ and $\delta^{15}N$ human values from the LdM phases 2-5, Amazon basin, Classic Maya and early maize Maya sites **18**

Table S4 Description of the $\delta^{13}C$ and $\delta^{15}N$ muscovy duck values from Salvatierra and Panama **19**

Table S5 Long term average of the SRMs used in this study. **19**

Table S6 Mean and standard deviation of all check and calibration standard reference materials **20**

Table S7 Results of the Shapiro-Wilk normality tests **20**

Site context, chronologies and funerary practices

The monumental mound sites (formerly called Lomas) analyzed in this study are located in the village of Casarabe, about 50km to the east of Trinidad, in the Bolivian province of El Beni (Figures 1 and S4, 14° 52' 13.76" S, 64° 28' 48.65" W). Despite initial excavations at Loma Alta de Casarabe[92,93], recent advances of the project "Lomas de Casarabe" led by Heiko Prümers provided a fundamental contribution with the excavation of Mendoza and Salvatierra (Figures S6 and S7). The efforts of Prümers and his team have provided the most extensive and detailed evidence of the chronologies, pottery remains, burial practices, animal and plant remains of all excavated mound sites in the LdM[11,13,57,58,69].

Mendoza site

Mendoza (Figure S6) was the first site excavated by the Lomas de Casarabe project with the primary intention of better understanding the local chronologies and material culture. This initial effort provided important information about the mound construction, showing the initial development of a shallow platform built on top of a paleoriver bank into a 6m high earthen structure. The constructed mound area covers about 3.5 hectares, reaching over 6m above the ground. The construction of a recent road across the mound, splitting it into two halves, allowed the research team to expose a 76m long profile on the bluff to the west of the road. The highest remaining portion and the southwestern corner were also excavated, encompassing the paleoriver next to the mound. Extensive radiocarbon dating (n=46, Figure S1) shows the mound was continuously constructed and occupied from 500 to 1400 CE.

The material culture recovered includes mostly ceramic vessels of varying sizes and decoration types – painting, incisions and appliqués – with more complex geometric patterns appearing later in the sequence. In addition to vessels, ceramic hand rollers, figurines, and decorated spindle whorls were common finds. Lithic tools include only a few polished stone axes, interpreted as imports given the absence of stone in the Mojos alluvial plains[94].

In total 60 human burials were excavated at Mendoza site; however, only 28 were in areas with a clear deposition context while the other 32 could not have their chronologies defined. The remains had their age, sex and phase context (detailed in Supplement 2) determined by Trautmnn and colleagues [84]. Most burials were recovered in excavations 9, 1 and 2, with a single individual from excavation 5 (Figure S6). There is only one burial context dated at Mendoza, (No. 121 dated to 1275±37 cal. years BP, see list in 56: fig. 105), but according to the deposition contexts, most burials belong to phases 3 and 4. During phase 4, most burials (n=17) were found inside large and ornate ceramic burial urns, some containing funerary accompaniments such as beads and shell necklaces (LM56, LM121).

Salvatierra site

After the local contexts and chronologies were determined at Mendoza, the project's efforts were turned towards the Salvatierra site, located about 4 km to the west of Mendoza (Figure S5). Much like Mendoza, Salvatierra (Figure S7) was also built next to a paleoriver, taking advantage of the high embankment formed in the inner part of a meander to construct an expansive terrace of about 4ha, raised 1.5m above ground level. Atop the terrace area, there are two taller mounds (named 1 and 2). Mound 1 is the larger ‘U’ shaped mound located to the northeast of the terrace area, covering about 1 hectare in size and rising 7 m above the ground level. Mound 2 is smaller, approximately 40x40m and 5m high, connected via a platform to the southwest of Mound 1. Surrounding the wider terrace is a polygonal ditch connected via a canal to the seasonally flooded savannah to the south (possibly defensive), where two circular reservoirs and a dam were also constructed, interpreted as water management systems[27]. The whole area covered by landscape transformation at Salvatierra, including mounds, ditches, canals and reservoirs, extends for about 27ha[27]. Excavations of the site focused chiefly on the two mound areas: Mound 1 (184m^2 - 23x8m - of excavations) and Mound 2 (200 m^2 of excavations, including a dense funerary context) (Figure S7). Two other areas were excavated in

the terrace portion of the site – unit 2 (6x8 m) and unit 12 (6x7 m) – where large quantities of ceramic remains were recovered[57].

An additional 48 radiocarbon dates show the same occupation period as Mendoza, between 500 and 1400 CE (Figure S2), which, given the similarities and abundance of pottery remains, led Jaimes Betancourt[57] to divide both sites into five distinct ceramic phases (Figures S1 and S2). Aside from the abundance of ceramic remains, large quantities of plant[11,13] and animal remains were also recovered at Salvatierra[69].

The Salvatierra site also contains what is possibly the largest assemblage of human burials ever excavated from a single site in the whole of the Amazon basin, with 123 individuals recovered. Overall, the human remains are well-preserved, often articulated and mostly complete. Of the total assemblage, only 90 individuals could be characterized according to the ceramic phases, comprising phases 2 through 5 (700-1400 CE).

Burial practices show a variety of body placements, with a prevalent NW/SE (head/feet) orientation, rare funerary accompaniments, but also a few uncommon contexts. In all phases, a few individuals are buried with complete ceramic vessels deposited over or near the individuals' heads. In the later phases, 4 and 5, a few individuals are buried facing down or in a seated position. Much like Mendoza, there is also the late appearance of urn burials during phases 4 and 5, largely comprised of new-borns and infants (see 95:20-23).

Burials were found in all ten areas excavated in the mound area, with marked differences between the terrace area, Mounds 1 and 2. While the terrace area (interpreted as domestic) and Mound 1 (interpreted as ceremonial/political) have low burial densities, Mound 2 seems to be a dedicated funerary area, with the highest burial concentration in the site encompassing almost fifty individuals in an excavated area of 200 square meters[27].

Mound 2 also had the most impressive burial recovered from Salvatierra, pertaining to phase 2. In its very centre, at around 3m deep, Burial 1005 (1341±24 cal. years BP, KIA 31855) included remains of a single adult male, aged between 30 and 40 years old, placed in an extended

position with an extraordinary quantity and quality of funerary accompaniment[85,96]. The body was dressed in several necklaces containing over 5000 shell beads – found still aligned in at least seven strands – complemented by four jaguar canines and three large blue stone beads. On the left wrist, there was a large bracelet made of 47 polished bone pieces, all found aligned, wrapped around his forearm. Over the forehead and on both sides of the head there were three large copper disc (from the Andes), likely part of a headdress, as well as a lip plug (tembetá) made of Amazonite (likely from Brazil). This individual also had the highest $\delta^{13}C$ value of all adults in the assemblage which, under the uncommonly high quantity and quality of burial accompaniments – including pieces of copper metal brought from the Andes, as there is no evidence of metallurgy in the SA Lowlands – indicate an equally uncommon social status, hinting at a political and/or religious importance.

It is worth noting that burial 1218a is a 9 month old individual with the lowest $\delta^{15}N$ value of the whole assemblage sampled in this study (3.6‰, mean Salvatierra $\delta^{15}N$ 9.0±1.7) indicating a diet based largely on plants, thus suggesting a potential severe state of malnutrition at the time of death. The contextual unreliability of the obtained results justify its removal from the Bayesian inference in Figure 3B, as it does not represent the population of Phase 5, not to mention the inherent paradox in drawing dietary information from a non-surviving infant whose premature death was likely the consequence of poor nutrition[97.98].

Fauna

Like the macrobotanical remains, Loma Salvatierra is the only site in the Casarabe area where faunal remains have been studied. Remains include a variety of mammals, reptiles, amphibians, birds, fish and molluscs[69]. It is worth noting that all taxa had their number of recovered fragment reported but only mammals and fish had the total mass of recovered fragments by taxa recorded.

Mammals comprise the most common bone remains, representing 49% of all identified bone fragments and over 92% of all recovered fauna remains by weight. In total, 24 species belonging to six families were identified. Results show a clear dominance of Cervidae remains, with four identified species – red brocket deer, *Mazama americana,* grey brocket deer, *M. gouazoubira*; marsh deer, *Blastoceros dichotomus;* and white-tailed deer, *Odocoileus virginianus* – making up to 75% of all mammal bones by mass and over 50% of all identified fragments. Other mammals such as rodents (*Myocastor coypu, Agouti paca* and *Dasyprocta variegata s. punctata*) represent around 12% or recovered fragments by mass, including only 4 capibara fragments identified; while armadillos (*Dasypus novemcinctus* and *Euphractus sexcinctus*) encompass only 2.5% of recovered fragments by weight.

Fish are the second most representative taxon, containing 35% of identified animal bones but only around 7.5% of the recovered remains by weight. Fish are composed mainly of three families of eel – Synbranchideae, Callichthyidae and Lepidosirenidae – that combined make up to 95% of all fish remains[99].

Birds are the third most representative taxon with 7.5% of all identified fragments. Bird remains are dominated by muscovy duck (*Cairina moschata*), constituting 90% of the bird remains. There are no data regarding their representation by mass; however the small size of ducks (~4kg for modern domesticated) in comparison to more abundant fauna such as deer (~30kg) indicates they likely had a representation similar to that of fish.

As for the faunas' dietary sources, the intermediate $\delta^{13}C$ values found in the riverine taxa and rodents likely reflects the local balance between C_3 and C_4 plants, although maize is not excluded as a potential contributor. As for the armadillos, their stable isotope values vary considerably between a variety of studies[48, 70, 71, 100]. Reasons for this phenomena are still unknown (perhaps due to their generalist diets often including an assortment of invertebrates), but they tend to have more elevated $\delta^{13}C$ values than other local taxa[70], even in pre-maize contexts [71,100].

Two deer samples (SAF50 and SAF56 in Supplement 2) were not included in the Baysian inference in Figure 2 and other statistical comparisons. These samples showed elevated $\delta^{13}C$ values indicating the consumption of different levels of C_4 plants, meaning they were either remains of marsh deer (*Blastocerus dichotomus*), known to graze C_4 grasses, or maize consuming brocket deer. These samples were removed from the analyses as the objective of the Ungulate group is to provide a baseline for exclusive C_3 plant consumers in the region. Further studies are needed to verify the consumption or maize in the brocket deer population during the Casarabe culture. Similarly, the only two muscovy duck coracoid fragments analysed (SAF 39 and SAF 61) were not included in the Bayesian inference in Fig. 2 as these samples displayed a number of issues. These were very small (<1g) bone fragments, leading to possible misidentification, and showed very similar $\delta^{13}C$ and $\delta^{15}N$ values between themselves despite being from different phases and excavation units. The $\delta^{13}C$ values obtained for these two samples diverge by about +6‰ when compared to the rest of the duck population. These values are more similar to the Rodent and Riverine groups, likely reflecting the local balance of C_3 and C_4 sources available in the wild. This body of evidence led us to conclude these were likely misidentified remains, potentially fragments of other duck species (*Anas* cf. *specularioides*) found at Salvatierra[69]. As these could also be wild muscovy duck, the values were included in the comparison with archaeological domesticated muscovy duck from Panama presented in Fig. S4 and Table S4.

References

92. Dougherty, B. & Calandra, H. Prehispanic human settlement in the Llanos de Moxos, Bolivia. In *Quaternary of South America and Antarctic Peninsula* (ed Jorge Rabassa) 163-99 (CRC Press, London, 1984).

93. Dougherty, B. et al. Excavaciones em la Loma Alta de Casarabe, Departamento del Beni, Bolivia. *Rel. Soc. Arg.Antropol.* **14**, 9-48 (1982).

94. Prümers, H. The Andes as seen from Mojos. In: A. J. Pearce, D. G. Beresford-Jones, and P. Heggarty (eds.), *Rethinking the Andes Amazonia Divide: A Cross-Disciplinary Exploration.* pp. 263-272. London: UCL Press. (2020)

95. Prümers, H. & Jaimes Betancourt, C. 100 años de investigación arqueológica en los Llanos de Mojos. *Arqueoantropológicas* **4**, 11–53 (2014).

96. Prümers, H. `Charlatanocracia´ en Mojos? Investigaciones arqueológicas en la Loma Salvatierra, Beni, Bolivia. *Bol. Arqueol. PUCP* **11**, 103-116 (2007).

97. Wood, J.W. , Milner, G.R., Harpending, H.C., Weiss, K.M. The osteological paradox: problems of inferring prehistoric health from skeletal samples [and comments and reply]. *Curr. Anthropol.* ***33***, 343-370 (1992). [https://doi.org/10.1086/204084]

98. Wright, L.E., & Yoder, C.J. Recent progress in bioarchaeology: approaches to the osteological paradox. *J. Archaeol. Res.* **11**, 43-70 (2003).

99. Prestes-Carneiro, G., Béarez, P., Shock, M. P., Prümers, H. & Jaimes Betancourt, C. Pre-Hispanic fishing practices in interfluvial Amazonia: zooarchaeological evidence from managed landscapes on the Llanos de Mojos savanna. *PLoS ONE* 14, e0214638 (2019).

100. Hermenegildo, T. *Reconstituição da dieta e dos padrões de subsistência das populações pré-históricas de caçadores-coletores do Brasil Central através da ecologia isotópica* (University of São Paulo, unpublished master's thesis, 2009).

101. Sugiyama, N., France, C. A., Cooke, R. G., & Martínez-Polanco, M. F. Collagen and carbonate isotope data of fauna from pre-Columbian Panama. *Data Brief*, ***31***, 105974. (2020).

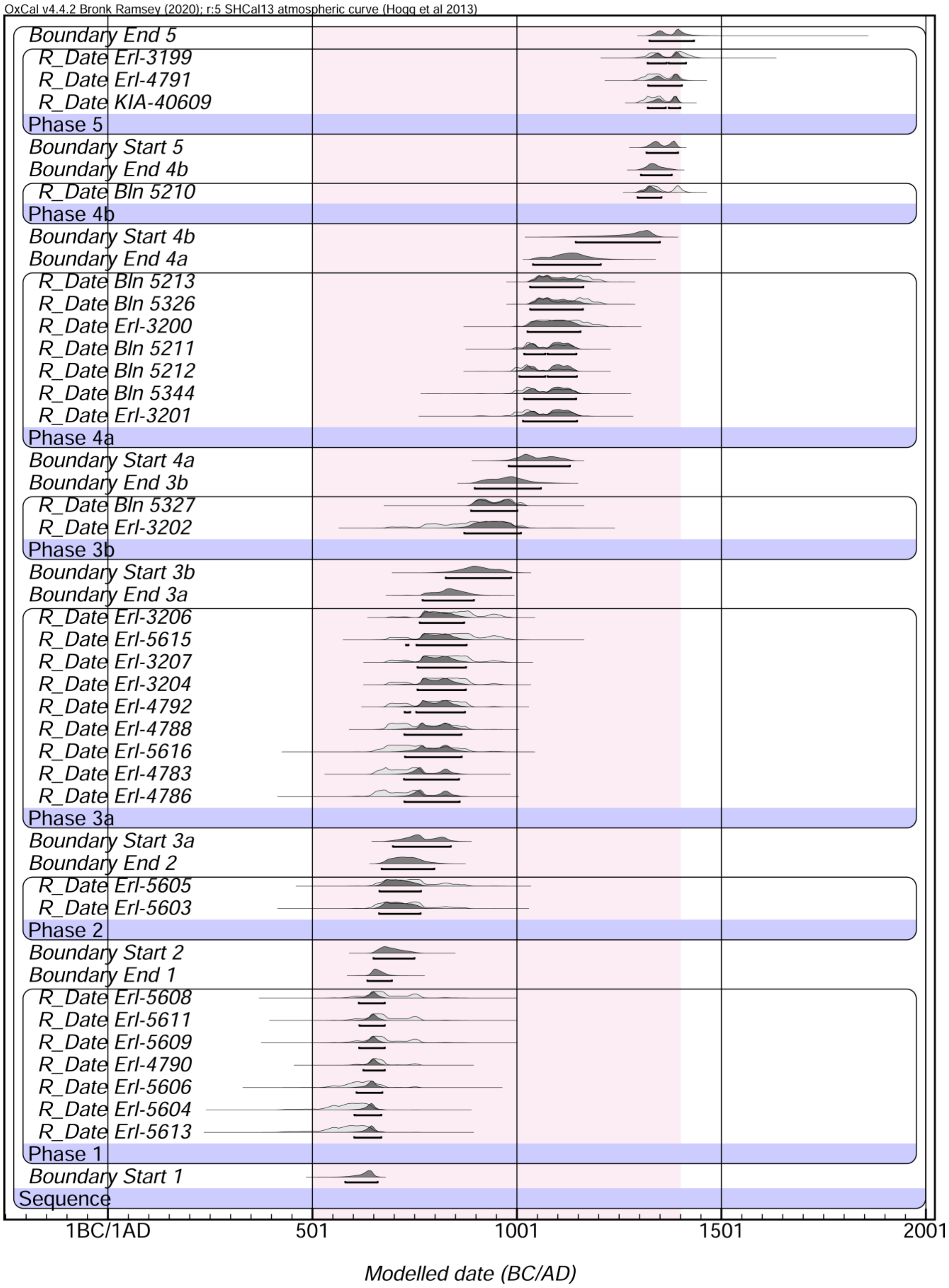

Figure S1 Radiocarbon Dates from Mendoza site arranged by phase. Outliers and samples from sterile soil underlying the mound were excluded. From 27, supplementary figure 4.

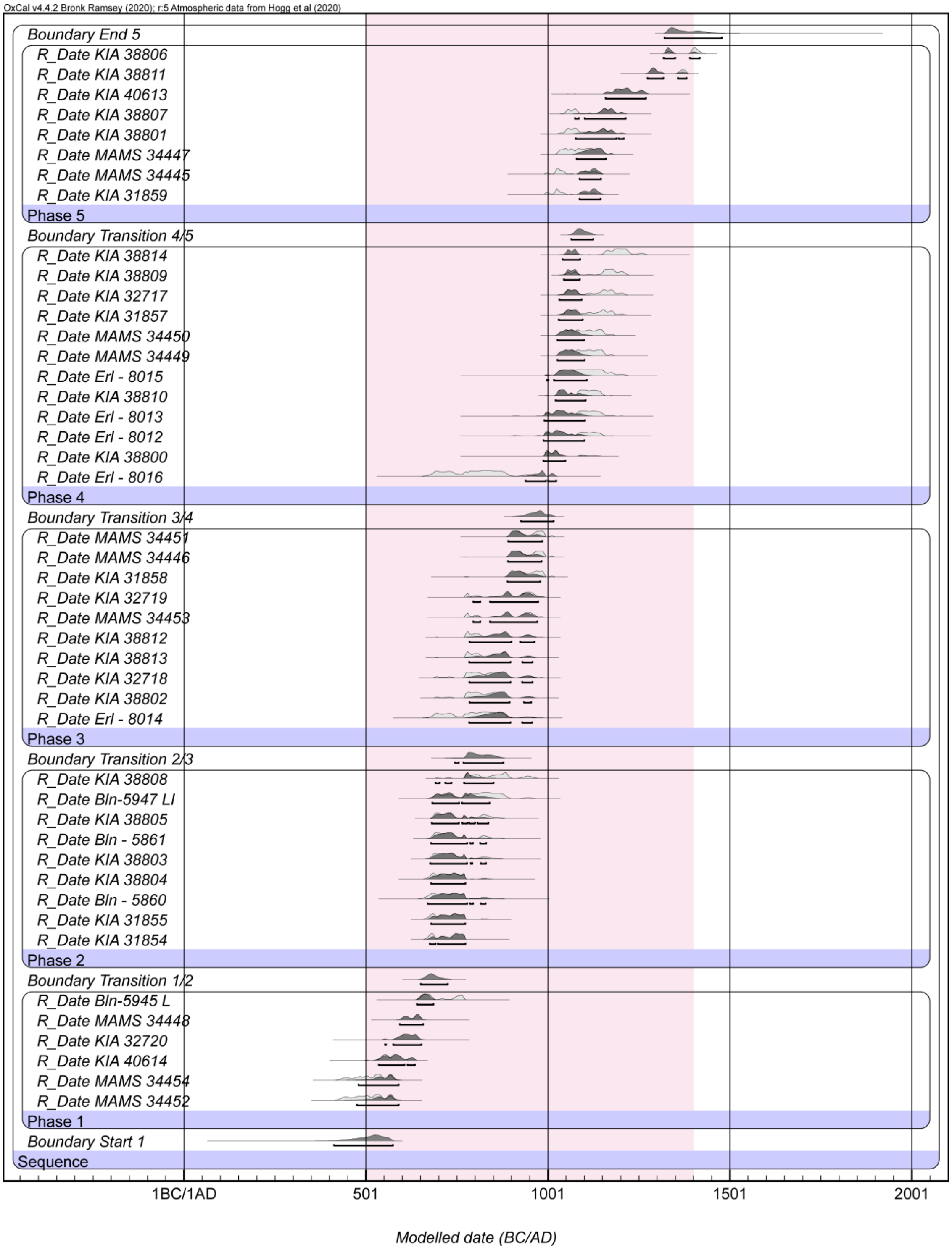

Figure S2 Radiocarbon Dates from Salvatierra site arranged by phase. Outliers and samples from sterile soil underlying the mound were excluded. From 27, supplementary figure 3.

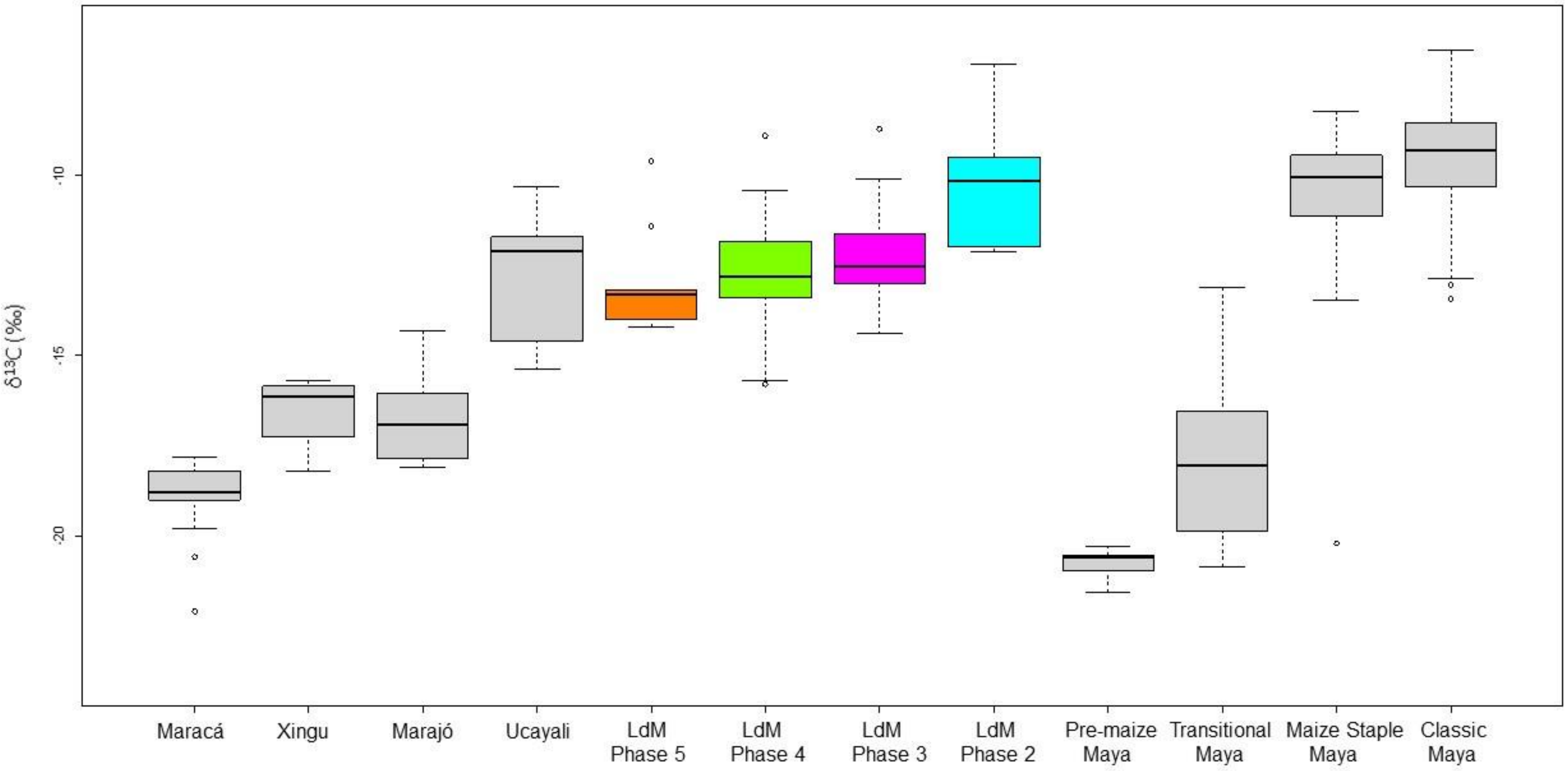

δ13C (‰)
-10
-15
-20
Maracá
Xingu
Marajó
Ucayali
LdM Phase 5
LdM Phase 4
LdM Phase 3
LdM Phase 2
Pre-maize Maya
Transitional Maya
Maize Staple Maya
Classic Maya

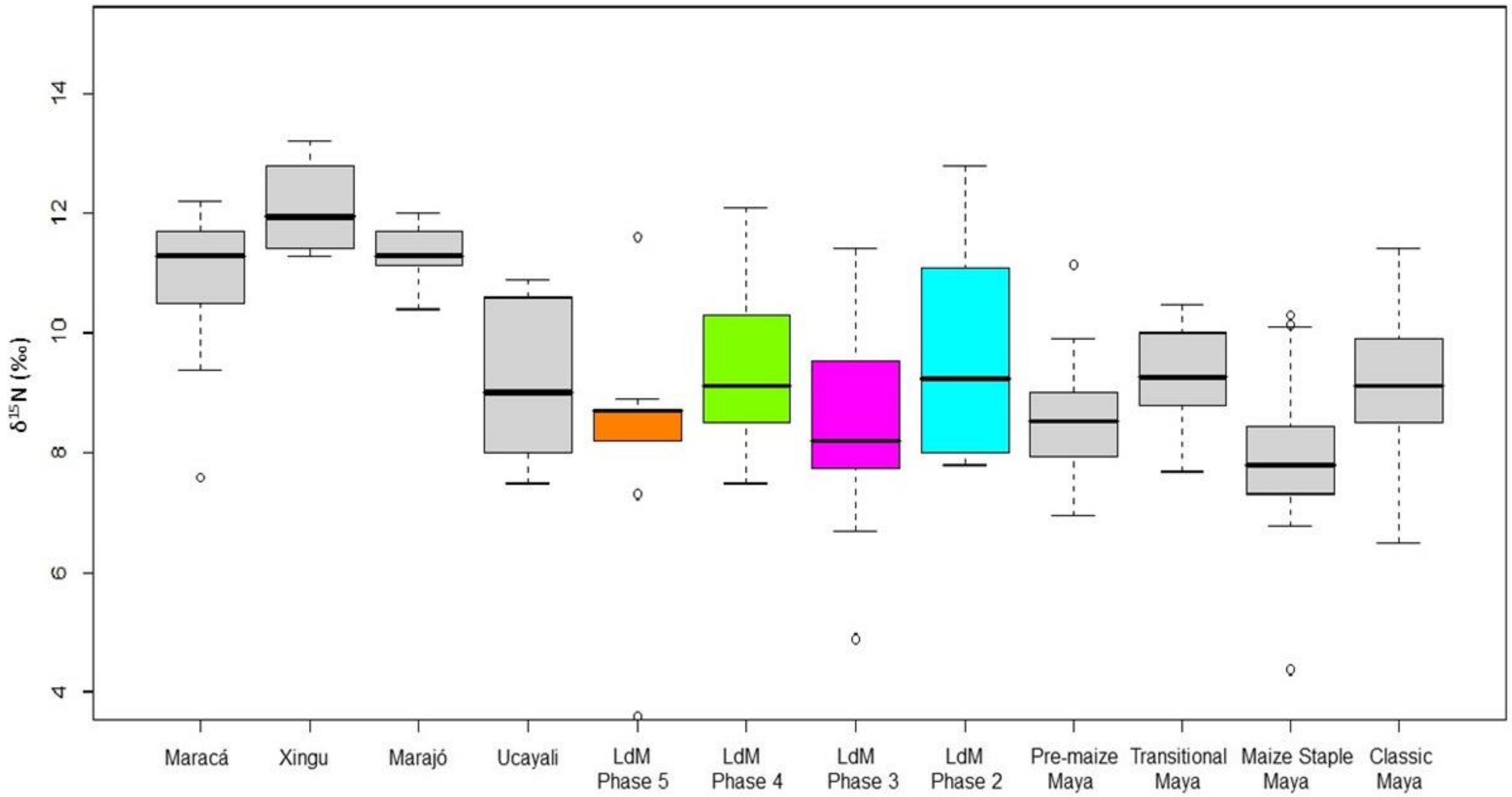

Figure S3 Boxplots presenting the $\delta^{13}C$ (above) and $\delta^{15}N$ values (below) of the humans from sites in the Amazon basin (Maracá, n=17; Xingu, n=4; Marajó, n=7; and Ucayali n=9), the Llanos de Mojos (highlighted, phase 2, n=10; phase 3, n=27; phase 4, n=27; and phase 5, n=9), pre-maize (n=16, 7600-2700 BCE), transitional maize (n=11, 2700-2000 BCE) and maize staple diets (2000 BCE-1000 CE) in Belize (n=25), as well as values from Classic Maya sites from Guatemala (n=130). Horizontal lines represent median and hinges the 25th (Q1) and 75th percentiles (Q3). Whiskers correspond to the larget and smallest observations (±1.5*IQR) while circles represent outlyers (>1.5*IQR and <-1.5*IQR). Data from 43, 44, 54, 55 and 60-62, values in Table S3.

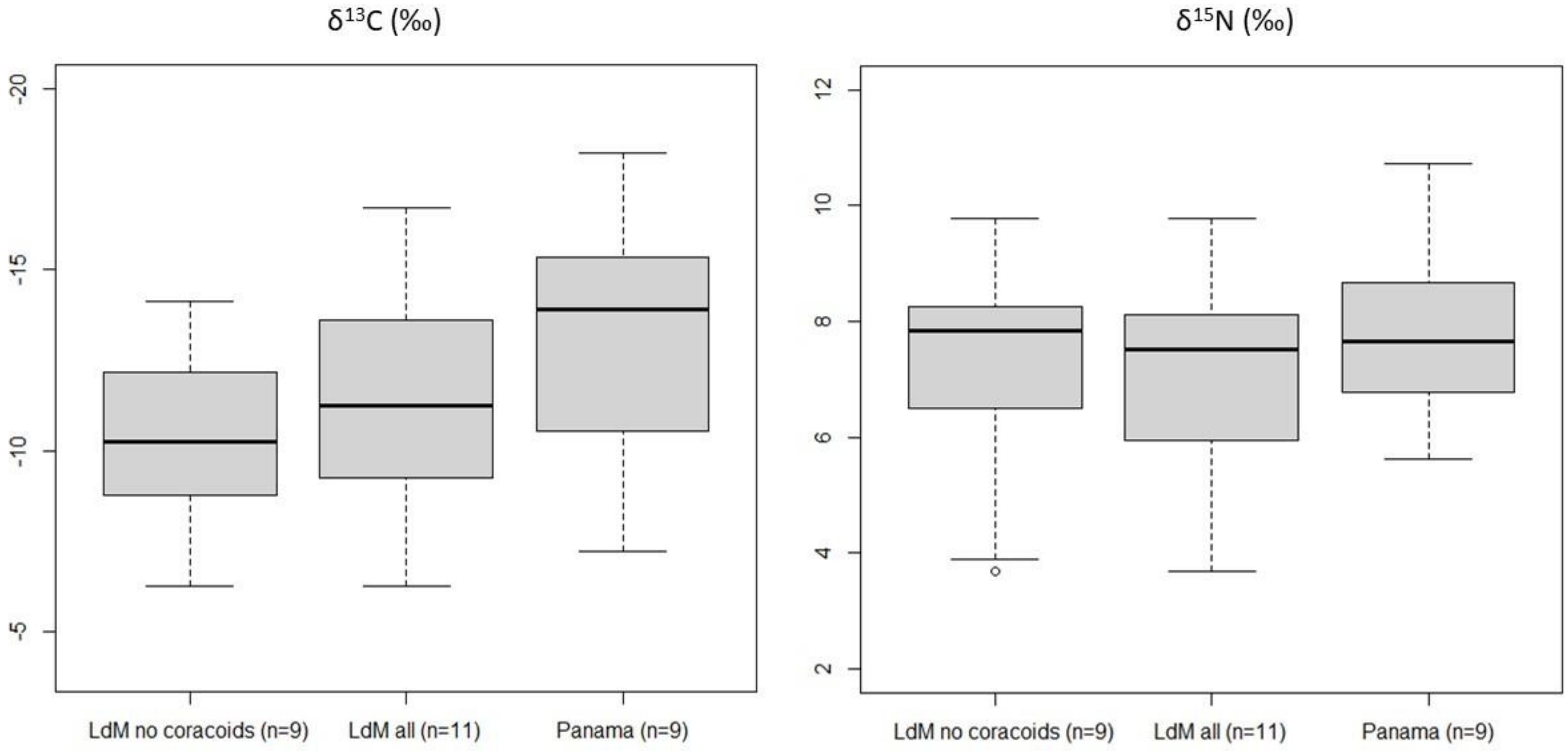

Figure S4 Boxplots presenting the $\delta^{13}C$ and $\delta^{15}N$ values of the muscovy duck population from Salvatierra (with and without potentially misidentified coracoid samples) and Panama sites [72,100]. Horizontal lines represent median and hinges the 25th (Q1) and 75th percentiles (Q3). Whiskers correspond to the larget and smallest observations (±1.5*IQR) while circles represent outlyers (>1.5*IQR and <-1.5*IQR). Data from 101.

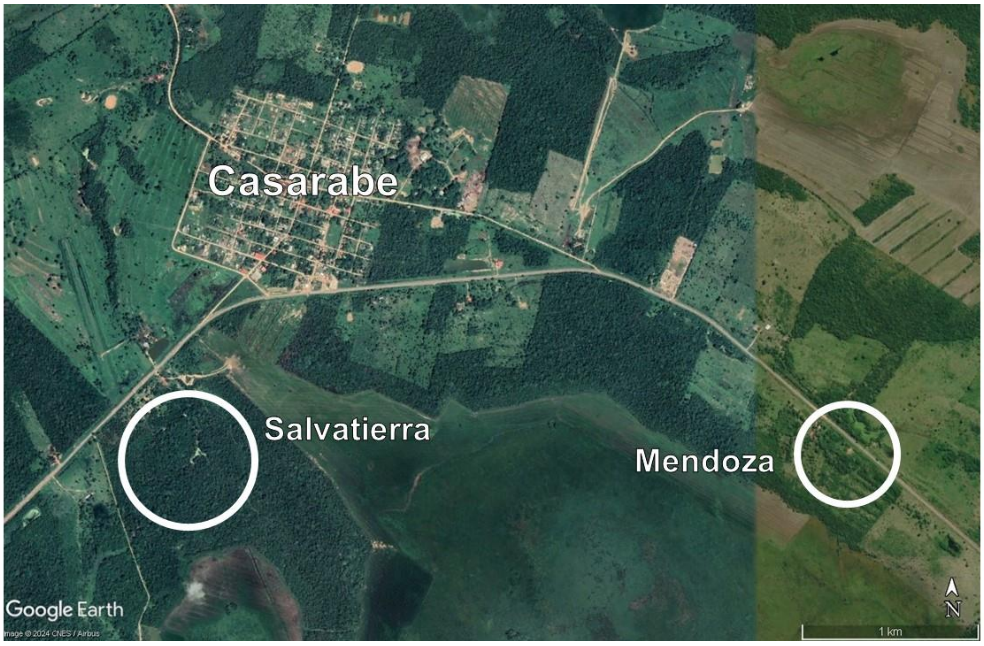

Figure S5 Aerial view from the village of Casarabe, Bolivia (14° 52' 13.76" S, 64° 28' 48.65" W), showing the proximity between the studied sites. Map data Google ©2024 CNES/Airbus.

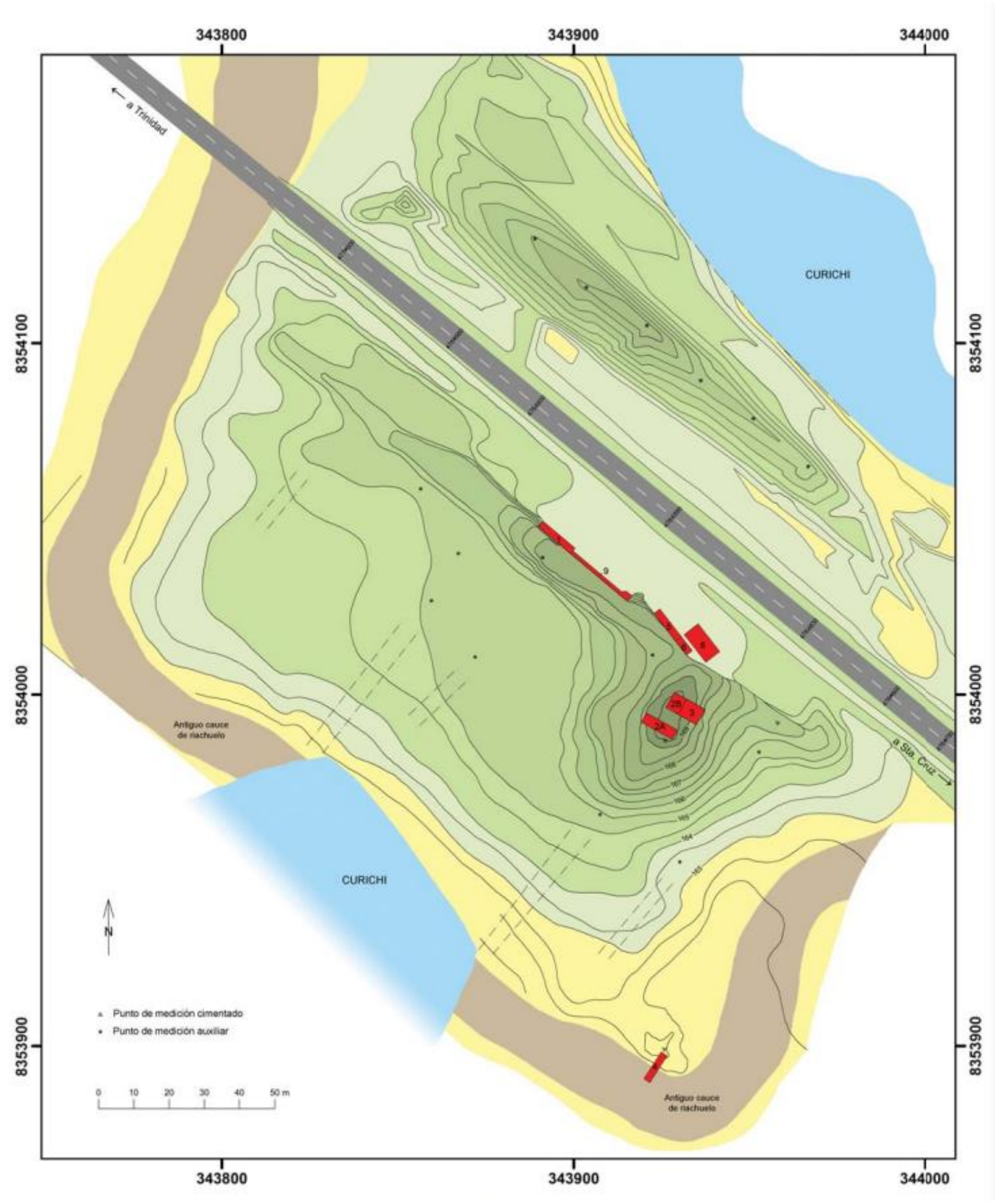

Figure S6 The Mendoza site plan, with each of the nine excavated areas highlighted in red. Graphic by H. Prümers.

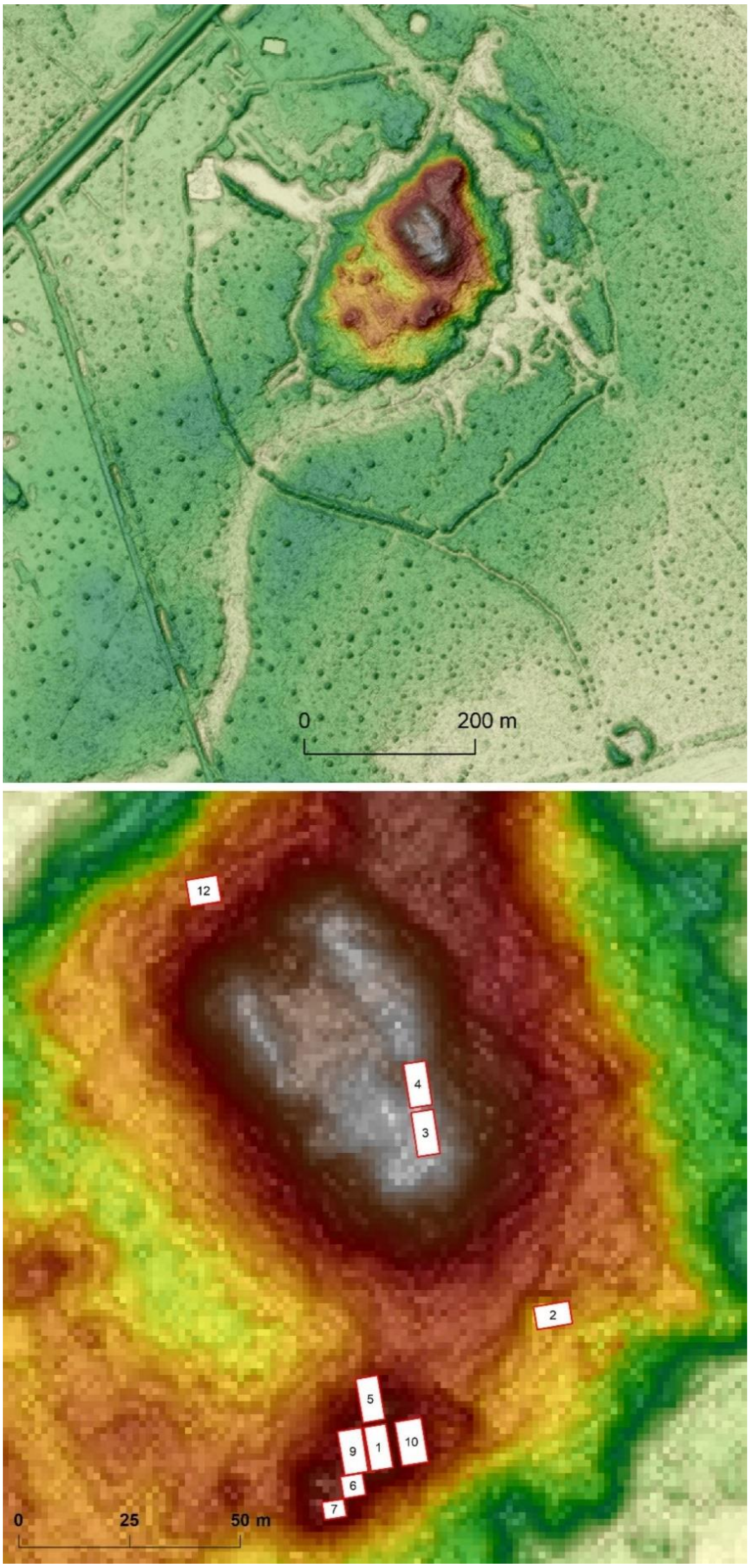

Figure S7 The Salvatiera site plan. Top: LIDAR reconstruction of the site are showing tis main features. Bottom: detail showing the wider plateau area (dark red/brown), the U shaped Mound 1(north) and Mound 2 (south), and the units excavated by the Lomas de Casarabe project (red). Graphic by H. Prümers.

Table S1 Results of post-hoc pairwise comparisons (p values) of the $\delta^{13}C$ (Tukey's HSD test, two sided) and $\delta^{15}N$ (Dunn's test, two sided) fauna values. α=0.05. Ungulates n=10, rodents n=8, armadillos n=14, muscovy duck n=9, riverine (eels and caimans) n=19. No adjustments were made for multiple comparisons.

**$\delta^{13}C$ (‰)**				
	Ungulates	Rodents	Armadillos	Muscovy duck
Rodents	5.42×10^{-10}			
Armadillos	2.59×10^{-11}	0.0030		
Muscovy duck	2.59×10^{-11}	0.0335	0.9781	
Riverine	0.00004	0.0004	2.63×10^{-11}	4.29×10^{-10}
**$\delta^{15}N$ (‰)**				
	Ungulates	Rodents	Armadillos	Muscovy duck
Rodents	0.0396			
Armadillos	0.0002	1.59×10^{-8}		
Muscovy duck	0.0962	0.0003	0.0718	
Riverine	0.7438	0.0088	0.00006	0.1183

Table S2 Results (p values) of the Tukey HSD pairwise comparisons (two sided) test of the human $\delta^{13}C$ values according to occupation phase. α=0.05. Phase 2 n=10, phase 3 n=27, phase 4 n=27, and phase 5 n=9.

	**Phase 2**	**Phase 3**	**Phase 4**
**Phase 3**	0.003067		
**Phase 4**	0.0001895	0.6778	
**Phase 5**	0.001331	0.6613	0.9845

Table S3 Description of human carbon and nitrogen isotopic values from LdM phases 2-5, the Amazon basin, Classic Maya sites and early maize Maya sites. *values in parenthesis represent all phase 5 individuals except LS1218a, n=8. Data source: Xingu 44, Maraca 43, Marajó 55, Ucayali 54, Altar de Sacrificios and Seibal 60 and 61, Holmul 61, Ealy maize Maya 62.

**Llanos de Mojos**								
	Phase 2 (n=10)		Phase 3 (n=27)		Phase 4 (n=27)		Phase 5* (n=9)	
	$\delta^{13}C$ (‰)	$\delta^{15}N$ (‰)	$\delta^{13}C$ (‰)	$\delta^{15}N$ (‰)	$\delta^{13}C$ (‰)	$\delta^{15}N$ (‰)	$\delta^{13}C$ (‰)	$\delta^{15}N$ (‰)
**Mean**	-10.2	9.7	-12.3	8.5	-12.7	9.4	-12.9(-13.4)	8.3(8.8)
**1σ**	1.8	1.8	1.3	1.5	1.6	1.3	1.5(0.9)	2.1(1.2)
**Range**	5.2	5.0	5.7	6.5	6.9	4.6	4.6(2.8)	8.0(4.3)
**Median**	-10.2	9.5	-12.5	8.2	-12.8	9.1	-13.3(-13.3)	8.7(8.7)
**IQR**	2.9	3.4	1.5	2.0	1.6	1.9	1.8(0.9)	1.1(0.6)
**Amazon basin**								
	Xingu (n=4)		Maracá (n=17)		Marajó (n=7)		Ucayali (n=9)	
	$\delta^{13}C$ (‰)	$\delta^{15}N$ (‰)	$\delta^{13}C$ (‰)	$\delta^{15}N$ (‰)	$\delta^{13}C$ (‰)	$\delta^{15}N$ (‰)	$\delta^{13}C$ (‰)	$\delta^{15}N$ (‰)
**Mean**	-16.6	12.1	-19.0	11.4	-16.7	10.1	-12.9	9.3
**1σ**	1.1	0.9	1.1	0.5	1.4	1.4	1.8	1.3
**Range**	2.5	1.9	4.3	1.5	3.8	4.6	5.1	3.4
**Median**	-16.2	12.0	-18.8	11.5	-16.9	10.4	-12.1	9.0
**IQR**	0.8	1.1	1.1	0.7	2.1	1.1	3.2	2.7
**Classic Maya**								
	Altar de Sacrificios (n=55)		Seibal (n=61)		Holmul (n=14)		All (n=130)	
	$\delta^{13}C$ (‰)	$\delta^{15}N$ (‰)	$\delta^{13}C$ (‰)	$\delta^{15}N$ (‰)	$\delta^{13}C$ (‰)	$\delta^{15}N$ (‰)	$\delta^{13}C$ (‰)	$\delta^{15}N$ (‰)
**Mean**	-9.3	8.7	-9.5	9.5	-9.4	9.2	-9.4	9.1
**1σ**	1.2	1.0	1.2	0.9	1.3	0.8	1.2	1.0
**Range**	6.9	5.0	5.4	4.2	3.8	3.3	6.9	5.0
**Median**	-9.1	8.7	-9.3	9.6	-9.1	9.3	-9.3	9.1
**IQR**	1.3	1.2	1.7	1.1	2.1	0.9	1.7	1.3

**Early maize Maya**						
	Pre maize (n=16)		Transitional maize (n=11)		Maize staple (n=25)	
	$\delta^{13}C$ (‰)	$\delta^{15}N$ (‰)	$\delta^{13}C$ (‰)	$\delta^{15}N$ (‰)	$\delta^{13}C$ (‰)	$\delta^{15}N$ (‰)
**Mean**	-20.8	8.6	-17.9	9.3	-10.7	8.0
**1σ**	0.4	1.0	2.5	0.9	2.3	1.3
**Range**	1.3	4.2	7.7	2.8	12.0	5.9
**Median**	-20.6	8.5	-18.1	9.3	-10.0	7.8
**IQR**	0.5	1.2	3.8	1.5	1.9	1.2

Table S4 Description of the muscovy duck carbon and nitrogen isotopic values from Salvatierra (with and without potentially misidentified coracoid samples) as well as results from Panama sites. Data from 97.

**Muscovy duck**						
	Salvatierra no coracoids (n=9)		Salvatierra all (n=11)		Panama (n=9)	
	δ^{13}C (‰)	δ^{15}N (‰)	δ^{13}C (‰)	δ^{15}N (‰)	δ^{13}C (‰)	δ^{15}N (‰)
**Mean**	-10.3	7.2	-11.5	7.0	-13.1	7.8
**1σ**	2.6	2.1	3.5	2.0	3.7	1.6
**Range**	7.8	6.1	10.4	6.1	11.0	5.1
**Median**	-10.2	7.8	-11.2	7.5	-13.9	7.7
**IQR**	4.6	3.6	5.3	2.5	6.3	2.6

Table S5 Long term average of the standard reference materials (SRMs) used in this study. Values encompass four years previous to the analysis.

**International SRMs**			
**Standard**	**n**	**δ^{13}C (‰, VPDB)**	**δ^{15}N (‰, AIR)**
USGS40	634	-26.12 ± 0.07	-4.53 ± 0.10
IAEA600	1812	-27.49 ± 0.08	1.10 ± 0.08
**Laboratory SRMs**			
**Standard**	**n**	**δ^{13}C (‰, VPDB)**	**δ^{15}N (‰, AIR)**
Alanine	1864	-26.92 ± 0.08	-1.44 ± 0.08
Bovine Liver	821	-21.51 ± 0.15	7.63 ± 0.12
Nylon	1394	-26.28 ± 0.11	-1.60 ± 0.10

Table S6 Mean and standard deviation of all check and calibration standard reference materials (SRMs) for all analytical session containing data in this study. USGS40 standard made by IAEA.

**International SRMs**				
**Session**	**Standard**	**n**	**$\delta^{13}C$ (‰, VPDB)**	**$\delta^{15}N$ (‰, AIR)**
1	USGS40	9	-26.10 ± 0.07	-4.58 ± 0.04
2	USGS40	0	-	-
3	USGS40	9	-26.12 ± 0.02	-4.52 ± 0.05
4	USGS40	0	-	-
1	IAEA600	18	-27.56 ± 0.05	1.13 ± 0.04
2	IAEA600	27	-27.46 ± 0.05	1.15 ± 0.11
3	IAEA600	18	-27.49 ± 0.02	1.13 ± 0.07
4	IAEA600	18	-27.51 ± 0.03	1.12 ± 0.04
**Laboratory SRMs**				
**Session**	**Standard**	**n**	**$\delta^{13}C$ (‰, VPDB)**	**$\delta^{15}N$ (‰, AIR)**
1	Alanine	18	-26.89 ± 0.07	-1.45 ± 0.04
2	Alanine	18	-26.93 ± 0.04	-1.43 ± 0.05
3	Alanine	15	-26.92 ± 0.03	-1.46 ± 0.07
4	Alanine	18	-26.91 ± 0.07	-1.46 ± 0.06
1	Bovine Liver	9	-21.42 ± 0.09	7.62 ± 0.05
2	Bovine Liver	9	-21.55 ± 0.05	7.73 ± 0.05
3	Bovine Liver	9	-21.46 ± 0.06	7.68 ± 0.10
4	Bovine Liver	9	-21.57 ± 0.05	7.70 ± 0.04
1	Nylon	9	-26.05 ± 0.05	-1.68 ± 0.07
2	Nylon	18	-26.27 ± 0.09	-1.56 ± 0.09
3	Nylon	9	-26.30 ± 0.04	-1.61 ± 0.03
4	Nylon	18	-26.25 ± 0.06	-1.58 ± 0.06

Table S7 Results of the Shapiro-Wilk normality tests (one sided) for the different datasets compared in this study. α=0.05. ME = Mendoza site, SAL = Salvatierra site.

**Group**		**$\delta^{13}C$(‰)**		**$\delta^{15}N$ (‰)**	
	n	W	p	W	p
**Humans ME/SAL – All**	86	0.97	0.030	0.96	0.06
**Fauna**	64	0.972	0.1534	0.959	0.03241
**Humans ME/SAL – Phases 2-5**	73	0.9735	0.1269	0.9582	0.01631
