# Supplementary material for: Stable isotope evidence for pre-colonial maize agriculture and animal management in the Bolivian Amazon
Source: Nat Hum Behav. 2024 Dec 23;9(3):464–71. doi: 10.1038/s41562-024-02070-9 (PMC11936833; doi:10.1038/s41562-024-02070-9)
Supplement: Supplementary file 2 — Reporting Summary [file 41562_2024_2070_MOESM2_ESM.pdf]

nature portfolio

Corresponding author(s): Tiago Hermenegildo

Last updated by author(s): Sep 18, 2024

Reporting Summary

Nature Portfolio wishes to improve the reproducibility of the work that we publish. This form provides structure for consistency and transparency in reporting. For further information on Nature Portfolio policies, see our Editorial Policies and the Editorial Policy Checklist.

Statistics

For all statistical analyses, confirm that the following items are present in the figure legend, table legend, main text, or Methods section.

n/a	Confirmed	
☐	☒	The exact sample size (*n*) for each experimental group/condition, given as a discrete number and unit of measurement
☐	☒	A statement on whether measurements were taken from distinct samples or whether the same sample was measured repeatedly
☐	☒	The statistical test(s) used AND whether they are one- or two-sided<br>*Only common tests should be described solely by name; describe more complex techniques in the Methods section.*
☐	☒	A description of all covariates tested
☐	☒	A description of any assumptions or corrections, such as tests of normality and adjustment for multiple comparisons
☐	☒	A full description of the statistical parameters including central tendency (e.g. means) or other basic estimates (e.g. regression coefficient) AND variation (e.g. standard deviation) or associated estimates of uncertainty (e.g. confidence intervals)
☐	☒	For null hypothesis testing, the test statistic (e.g. *F*, *t*, *r*) with confidence intervals, effect sizes, degrees of freedom and *P* value noted<br>*Give P values as exact values whenever suitable.*
☒	☐	For Bayesian analysis, information on the choice of priors and Markov chain Monte Carlo settings
☒	☐	For hierarchical and complex designs, identification of the appropriate level for tests and full reporting of outcomes
☒	☐	Estimates of effect sizes (e.g. Cohen's *d*, Pearson's *r*), indicating how they were calculated

Our web collection on statistics for biologists contains articles on many of the points above.

Software and code

Policy information about availability of computer code

Data collection	Thermo Scientific Mass Spectrometry Software
Data analysis	R Core Team (2021); package SIBER (Jackson et al. 2011)

For manuscripts utilizing custom algorithms or software that are central to the research but not yet described in published literature, software must be made available to editors and reviewers. We strongly encourage code deposition in a community repository (e.g. GitHub). See the Nature Portfolio guidelines for submitting code & software for further information.

Data

Policy information about availability of data

All manuscripts must include a data availability statement. This statement should provide the following information, where applicable:

- Accession codes, unique identifiers, or web links for publicly available datasets
- A description of any restrictions on data availability
- For clinical datasets or third party data, please ensure that the statement adheres to our policy

All relevant data supporting this study are included in the article and the supporting materials

Research involving human participants, their data, or biological material

Policy information about studies with human participants or human data. See also policy information about sex, gender (identity/presentation), and sexual orientation and race, ethnicity and racism.

Reporting on sex and gender	N/A
Reporting on race, ethnicity, or other socially relevant groupings	N/A
Population characteristics	N/A
Recruitment	N/A
Ethics oversight	N/A

Note that full information on the approval of the study protocol must also be provided in the manuscript.

Field-specific reporting

Please select the one below that is the best fit for your research. If you are not sure, read the appropriate sections before making your selection.

☐ Life sciences ☒ Behavioural & social sciences ☐ Ecological, evolutionary & environmental sciences

For a reference copy of the document with all sections, see nature.com/documents/nr-reporting-summary-flat.pdf

Behavioural & social sciences study design

All studies must disclose on these points even when the disclosure is negative.

Study description	We studied the dietary composition of past human populations from the Amazon basin (Llanos de Mojos, Bolivia) based on quantitative stable isotope data (δ13C and δ15N) obtained from bone collagen.
Research sample	Samples include human and faunal bone remains recovered from two monumental mound archaeological sites, Salvatierra and Mendoza, dated to around 700-1400 CE. These sites were chosen as the have most detailed chronology and the largest representation of archaeological human remains recovered in the Llanos de Mojos region to date.<br>We analysed 159 bone samples of around 1g each. Human remains include individuals of all ages (0 - 65+) consiting of 24 individuals from Mendoza and 65 from Salvatierra.<br>Fauna had 70 samples from Salvatirerra collected for analysis, encompassing eleven distinct taxa of mammals, birds, reptiles and fish.
Sampling strategy	Sampling strategy was based on convenience, relying on archaeological bone material from the collection of the Commission for Archaeology of Non-European Cultures of the German Archaeologial Institute (KAAK-DAI) in Bonn, Germany. Currently the collection has been returned to Bolivia (Museo Kenneth Lee, Trinidad, Beni)<br>We collected human remains from all individuals available at the time of collection.<br>Fauna samples focused on covering the most representative taxa recovered at Salvatierra (around n=10 for each) in order to provide a baseline to which the human data can be interpreted. Fauna from Mendoza was not available for analysis.
Data collection	Archaeological bone material was excavated by Heiko Prumers (PI), Carla Jaimes Betancourt and team.<br>Bone collagen extraction and sample preparation was conducted by Tiago Hermenegildo at the Dorothy Garrod Laboratory for Isotopic Analysis, Department of Archaeology, University of Cambridge<br>The stable isotope analysis was carried out by Catherine Kneale, Mike Hall and James Rolfe at the Godwin Laboratory, Department of Earth Sciences, University of Cambridge.
Timing	Stable isotope analysis took two years. The analysis or experiments are not time dependent.
Data exclusions	Two deer samples (SAF50 and SAF56 in S2) were not included in the Baysian inference in Figure 2 and other statistical comparisons. These samples showed unusual δ13 C values indicating they were not C3 consumers . Further detail in S1
Non-participation	No participants were involved in this study
Randomization	Fauna groups were defined based on taxa (sometimes only to the level of genus since remains are often fragmented)<br>Human groups were divided according to occupation phases defined by the material culture (ceramic remains)

Reporting for specific materials, systems and methods

We require information from authors about some types of materials, experimental systems and methods used in many studies. Here, indicate whether each material, system or method listed is relevant to your study. If you are not sure if a list item applies to your research, read the appropriate section before selecting a response.

Materials & experimental systems

n/a	Involved in the study
☒	☐ Antibodies
☒	☐ Eukaryotic cell lines
☐	☒ Palaeontology and archaeology
☒	☐ Animals and other organisms
☒	☐ Clinical data
☒	☐ Dual use research of concern
☒	☐ Plants

Methods

n/a	Involved in the study
☒	☐ ChIP-seq
☒	☐ Flow cytometry
☒	☐ MRI-based neuroimaging

Palaeontology and Archaeology

Specimen provenance	Salvatierra and Mendoza sites, Casarabe, Beni Department, Bolivia
Specimen deposition	Museo Kenneth Lee (Archaeological Museum), Trinidad, Beni, Bolivia
Dating methods	No new dates are provided

☒ Tick this box to confirm that the raw and calibrated dates are available in the paper or in Supplementary Information.

Ethics oversight	The excavation and export of the archaeological material were approved by the Bolivian Vice-Ministry of Culture under UNAR AUT permit N° 026/02 for Mendoza and UNAR AUT permit N° 019/06 for Salvatierra.

Note that full information on the approval of the study protocol must also be provided in the manuscript.

Plants

Seed stocks	*Report on the source of all seed stocks or other plant material used. If applicable, state the seed stock centre and catalogue number. If plant specimens were collected from the field, describe the collection location, date and sampling procedures.*
Novel plant genotypes	*Describe the methods by which all novel plant genotypes were produced. This includes those generated by transgenic approaches, gene editing, chemical/radiation-based mutagenesis and hybridization. For transgenic lines, describe the transformation method, the number of independent lines analyzed and the generation upon which experiments were performed. For gene-edited lines, describe the editor used, the endogenous sequence targeted for editing, the targeting guide RNA sequence (if applicable) and how the editor was applied.*
Authentication	*Describe any authentication procedures for each seed stock used or novel genotype generated. Describe any experiments used to assess the effect of a mutation and, where applicable, how potential secondary effects (e.g. second site T-DNA insertions, mosiacism, off-target gene editing) were examined.*
